# Role of vitamin B_12_, folate, and thyroid stimulating hormone in dementia: A hospital-based study in north Indian population

**DOI:** 10.4103/0972-2327.74193

**Published:** 2010

**Authors:** Rachna Agarwal, Neelam Chhillar, Suman Kushwaha, Neeraj Kumar Singh, Chandra Bhushan Tripathi

**Affiliations:** Department of Neurochemistry, Institute of Human Behaviour & Allied Sciences, Delhi, India; 1 Department of Neurology, Institute of Human Behaviour & Allied Sciences, Delhi, India; 2 Department of Biostatics, Institute of Human Behaviour & Allied Sciences, Delhi, India

**Keywords:** Alzheimer disease, dementia, folate, thyroid stimulating hormone, vascular dementia, Vitamin B_12_

## Abstract

**Background:**

Vitamin B_12_ and folate represent modifiable risk factors for dementia. They may increase the risk of Alzheimer′s dementia (AD) and vascular dementia (VaD) as their deficiency can increase the homocysteine level due to slowed methylation reaction. Homocysteine has a neurotoxic effect that could lead to neurologic disturbances. Hence, it is important to explore the status of serum B_12_ and folate in AD and VaD to evolve the treatment strategies for the same.

**Objectives:**

A retrospective study was conducted to assess the levels of vitamin B_12_, folate, and thyroid stimulating hormone (TSH) in serum and the relationship of these factors, including age and sex to cognitive decline in VaD, AD, and dementia due to other causes (DOC).

**Materials and Methods:**

Serum vitamin B_12_, folate, TSH, and total cholesterol were studied in 32 AD patients (mean age: 65 years), 12 VaD patients (mean age: 61 years), 83 DOC (mean age: 65 years), and 127 control subjects (mean age: 49 years). Results: In AD, VaD, and DOC, the levels of vitamin B_12_ and folate were significantly lower (*P* < 0.002; 0.026; 0.002 for vitamin B_12_ and *P* < 0.000 in all the 3 groups for folate) as compared with the controls. Similarly, TSH levels were significantly lower in AD and DOC (*P* < 0.008; 0.038) as compared with the controls.

**Conclusion:**

Vitamin B_12_ and folate were significantly low in both AD and VaD patients. Hence, B vitamin supplementation should be considered as possible targets for the therapeutic intervention in dementia.

## Introduction

Dementia is one of the most frequent disorders among elderly patients, reaching to epidemic proportions with an estimated 4.6 million new cases worldwide each year.[1] With increasing life expectancy, the prevalence of dementia will increase dramatically in the next few decades, which will put immense burden on the health care system. Although dementia is an age-related disorder and an inevitable part of aging, attempts can be made to identify the nongenetic factors that may be modified to limit it. In various studies, deficiency of number of vitamins—B_12_, folic acid, and B_6_ —has been identified as candidate risk factor for both Alzheimer’s disease (AD) and vascular dementia (VaD).[2] Studies have shown lower serum B_12_ levels in subjects with AD and other dementias[3,4] and the effectiveness of vitamin B_12_ supplementation in improving cognition in demented patients.[5,6] However, such intervention studies found that the supplementation treatment was more effective in subjects with a short history of cognitive dysfunction. Similarly, the relationship between folate and AD or other dementia has also been explored. A number of cross-sectional studies have shown the relationship of low serum folate not only to AD[2,7] but all types of dementia.[8] Low folate levels have been found to be associated with a decline in cognitive functioning. Also, the positive effect of folate treatment has been found on memory deficits in a number of intervention studies.[9,10] The low blood levels of vitamin B_12_ and folic acid have been shown to increase the levels of homocysteine, which is recognized to be proatherogenic and prothrombotic and accepted as an independent vascular risk factor associated with AD and VaD.[11]

In recent studies, epidemiologic and pathologic evidence of an association between AD and indicators of atherosclerosis or presence of infarct have been reported.[12] Although the relationship between AD and vascular pathologies is unclear, studies illustrate that there may be a direct or additional contribution of cerebrovascular abnormalities[13,14] and lesions to cognitive impairment and to the pathogenesis and progression of AD.[15] Hence, there is a possibility that vascular risk factors might play a role in the pathogenesis of VaD as well as AD. Since 1969, hyperhomocysteinemia has been implicated in the pathogenesis of atherosclerosis[16] and is now considered as one of the modifiable independent risk factors for cardiovascular, peripheral vascular, and cerebrovascular disease.[17] Accordingly, a potential role for hyperhomocyteinemia in the etiology of AD has been postulated.[2,18] Variations in the levels of homocysteine have been shown to be due to nutritional status of folate, B_12_, and pyridoxine.[19] Deficiencies of these vitamins result in increased levels of homocysteine through diverse pathways.[20] Hence it is important to explore, whether there is a relationship of folate and vitamin B_12_ with dementia or not. It becomes more important to reveal the status of these vitamins in different types of dementia for 2 reasons. First, the levels of folate and vitamin B_12_ can affect the homocysteine status, which is not only a novel risk factor for cerebrovascular disease but also been implicated as a risk factor for AD and VaD. Second, if they have a role to play in the pathogenesis of dementia, vitamin therapy and dietary supplementation can reduce homocysteine levels and can be involved to help in developing the treatment strategies of dementia.[21,22]

In the current study, the levels of serum vitamin B_12_ and folate and their relationship to each other were studied in AD, VaD, and other dementia patients along with the nondemented controls. We also investigated other risk factors in different types of dementia, including thyroid function and lipid status. In the present study, the relationship of cognitive decline in dementia with these risk factors, including age and sex, have also been studied. Hence the aim of the study was to try to elucidate the possible similarities or differences between AD, VaD, and dementia due to other causes (DOC) in terms of levels of vitamin B_12_, folic acid, thyroid stimulating hormone (TSH), and total cholesterol (TC).

## Materials and Methods

A retrospective study was conducted by the Department of Neurochemistry in collaboration with the Department of Neurology in the Institute of Human Behavior and Allied Sciences, Delhi, India. The study was conducted in AD, VaD, patients with DOC, and control subjects.

Dementia patients (127; mean age: 64 years) and cognitive normal individuals (127; mean age: 49 years) were included in the study. Of the 127 patients, 32 patients with AD (17 men, 15 women; mean age: 65 years), 12 patients with VaD (09 men, 03 women; mean age: 61 years) and rest with DOC (48 men, 35 women; mean age: 65 years) were involved in the study. The diagnosis of probable AD was established following the criteria of the National Institute of Neurological and Communicative Disorders Association-Alzheimer’s Disease and Related Disorders Association (NINCDS-ADRDA).[23] The diagnosis was confirmed by cerebral magnetic resonance imaging (MRI) studies, which showed a generalized atrophy with selective temporoparietal atrophy in all the patients. The diagnosis of VaD was established according to the National Institute for Neurological Disorders and Stroke-Association Internationale pour la Recherché et l’Enseignement en Neurosciences (NINDS-AIREN) criteria for probable VaD. The diagnosis was confirmed by brain MRI showing microangiopathic pathology.

Elderly individuals (127; 58 men, 69 women; mean age: 49 years) attending the hospital with ailments other than cognitive impairment and coming to clinics routinely for other neurologic diseases were taken as the control group in the study.

All the patients with recent history of heart or respiratory failure, chronic liver or renal failure, malignant tumors, and recent history of alcohol abuse were excluded from the test and control group. Also subjects taking vitamin supplements were excluded from the study.

All the subjects were evaluated for Mini-Mental State Examination (MMSE) score. The mean values were 18.56 ± 0.88 in AD patients, 21.25 ± 0.95 in VaD, and 18.03 ± 0.45 in patients with DOC. All the subjects underwent clinical examination along with routine biochemical, hormonal, and radiologic examinations, including glucose, cholesterol, albumin, creatinine, thyroid hormonal assay, and MRI.

### Biochemical investigations

Nonfasting serum samples were obtained from patients taking all the standard precautions. The specimen was centrifuged within 30 min of sample collection to separate the serum and examined for routine biochemistry, including TC. Specimen for evaluation of vitamin B_12_, folate, and TSH was stored at –20°C until analysis. Precaution was taken to avoid exposure of the sample for vitamin B_12_ and folate to sunlight. Serum vitamin B_12_ and folate were measured by competitive immunoassay and TSH by using sandwich immunoassay technique. All these tests were performed on Electrochemiluminescence immunoassay analyzer, Elecsys 2010 (M/s Roche Diagnostics Asia Pacific Pte. Ltd, Singapore). The Coefficient of Variation (CV %) for vitamin B_12_, folate, and TSH were 10.96, 14.58, and 6.44 (normal values) and 10.44, 12.47, and 7.06 (higher values), respectively.

### Statistical analysis

Descriptive Statistics (mean and standard error) has been used to describe the data. Since all the variables are continuous in nature, one-way analysis of variance (ANOVA) technique was applied to find out the difference among the 4 groups (Controls, AD, VaD, and DOC) for the variables and if ANOVA showed a significant difference (*P* < 0.05), multiple comparison analysis was done by least significant difference (LSD) test to find out the significantly different group among these 4 groups. Correlation analysis was also done to establish the relationship between MMSE score and age, vitamin B_12_, folate, TSH, and TC in all the patients. All the analyses were done on Statistical Package for the Social Sciences (SPSS Version17.0, www.spss.com)

## Results

### Characteristics of the study population

Distribution and average with deviation of demographic and biochemical characteristics of study population is shown in Table 1. When compared for age, the control group was younger (48.65 ± 1.22 years) as compared with the dementia group (AD 65.03 ± 2.07, VaD 61.17 ± 3.03, DOC 64.52 ± 1.23 years). The controls were younger than 60 years (64%), whereas ony 30%–35% of all the 3 dementia groups were younger than 60 years. In the control group, 46% were male, whereas in AD 53%, VaD 42%, and DOC 58% were male. On an average, highest MMSE score (21.25 ± 0.95) was found in VaD group, whereas 96% subjects in the DOC group had MMSE score d 26. When vitamin B_12_ d 340 pg/mL was used to define low levels of vitamin B_12_,[24] 69% AD patients, 58% VaD, and 58% DOC patients had low vitamin B_12_ levels. Similarly, when low folate levels were defined as folate d 4.4 ng/mL,[24] among the different groups with dementia, 44% AD patients, 42% VaD patients, and 12% patients with DOC had low folate levels. The average level of TSH in the control group was 2.94 ± 0.23 mIU/L, whereas in the 3 dementia groups, that is, AD, VaD, and DOC mean TSH levels were 1.79 ± 0.19, 2.06 ± 0.28, and 2.3 ± 0.19 mIU/L, respectively. The TC level in the control group was 153.21 ± 2.36 mg/dL as compared with 187.18 ± 5.66, 182.75 ± 5.67, and 186.36 ± 2.68 mg/dL in AD, VaD, and DOC, respectively.

**Table 1:** Characteristics of study population

Characteristics	AD (32)	VaD (12)	DOC (83)
Age (years)			
Mean ± SE	65.03 ± 2.07	61.17 ± 3.03	64.52 ± 1.23
<60 (n)	10	04	29
≥60 (n)	22	08	54
Sex			
Male	17	05	48
Female	15	07	35
MMSE			
Mean ± SE	18.56 ± 0.88	21.25 ± 0.95	18.03 ± 0.45
≤26	29	09	80
>26	03	03	03
Vitamin B12 (pg/mL)			
Mean ± SE	266.59 ± 24.72	257.41 ±55.09	306.59 ± 15.92
≤340	22	07	48
>340	10	05	35
Folate (ng/mL)			
Mean ± SE	6.61 ± 1.15	6.30 ± 1.26	8.01 ± 0.47
≤4.4	14	05	10
>4.4	18	07	73
TSH (mIU/L)			
Mean ± SE	1.79 ± 0.19	2.06 ± 0.28	2.30 ± 0.19
≤ 1.3	13	02	30
>1.3	19	10	53
TC (mg/dL)			
Mean ± SE	187.18 ± 5.66	182.75 ± 5.67	186.36 ± 2.68
≤ 180	13	07	23
>180	19	05	60

AD, Alzheimer’s dementia; DOC, Dementia due to other causes; MMSE, Mini-Mental State Examination; SE, Standard error; TC, Total cholesterol; TSH, Thyroid stimulating hormone; VaD, Vascular dementia.

### Serum vitamin B_12_, folate, thyroid stimulating hormone, and total cholesterol levels in dementia groups

One-way ANOVA technique was applied to compare the levels of vitamin B_12_, folate, TSH, and TC levels in various dementia groups (AD, VaD, and DOC) and control and post hoc analysis was done by LSD test to eliminate the significantly different groups [Table 2]. AD group showed significantly lower levels of vitamin B_12_, folate, and TSH (*P* < 0.002, 0.000, and 0.008) as compared with the control, whereas in VaD, only vitamin B_12_ and folate levels were found to be significantly lower as compared with the controls (*P* < 0.026, 0.000). However, there was no difference in the levels of vitamin B_12_, folate, and TSH between AD and VaD groups. Similarly, in the DOC group, vitamin B_12_, folate, and TSH were significantly low as compared with the controls (*P* < 0.002, 0.000, and 0.038). However, TC levels were found to be significantly raised in all the groups of dementia, that is, AD, VaD, %DOC as compared with the controls (*P* = 0.000 in all).

**Table 2:** Statistical analysis of different parameters in various dementia groups

Parameters	Control (Mean ± SE)	AD (Mean ± SE)	VaD (Mean ± SE)	DOC (Mean ± SE)	Multiple comparison (LSD)		
					AD vs Control	VaD vs Control	DOC vs Control
Vitamin B_12_ (pg/mL)	170.19 ± 0.18	266.59 ± 24.72	257.41 ± 55.09	18.03 ± 0.45	0.002	0.026*	0.002*
Folate (ng/mL)	6.92 ± 1.18	6.61 ± 1.15	6.30 ± 1.26	8.01 ± 0.47	0.000†	0.000†	0.000†
TSH (mIU/L)	1.75 ± 0.18	1.79 ± 0.19	2.06 ± 0.28	2.30 ± 0.19	0.008	0.182	0.038*
TC (mg/dL)	191.05 ± 6.40	187.18 ± 5.66	182.75 ± 5.67	186.36 ± 2.68	0.000†	0.000†	0.000†

AD, Alzheimer’s dementia; DOC, Dementia due to other causes; LSD, Least significant difference; MMSE, Mini-Mental State Examination; SE, Standard error; TC, Total cholesterol; TSH, Thyroid stimulating hormone; VaD, Vascular dementia.

* Significant;

† highly significant.

### Correlation between mini-mental state examination scores and plasma variables

To determine the relationship of MMSE score with age and the plasma variables—vitamin B_12_, folate, TSH, and TC levels in dementia groups, the correlation analysis was performed between MMSE scores and the above-mentioned variables and is shown in Table 3. The MMSE scores were inversely and significantly related to age (*r* = –0.356, *P* = 0.001). Blood levels of vitamin B_12_ and folate also showed negative correlation with MMSE (*r* = –0.172, *P* = 0.0.05; *r* = –0.193, *P* = 0.03). However, TSH and TC did not show any correlation with MMSE (*r* = 0.075, *P* = 0.402 and *r* = –0.010, *P* = 0.916). The relationship was also examined separately in each group of dementia patients, namely, AD, VaD, and DOC. In AD patients, only vitamin B_12_ level and MMSE score were significantly and negatively correlated (*r* = –0.388, *P* = 0.028), whereas in VaD cases none of the variables were significantly correlated with the MMSE score. However, in the DOC group, age and folate were negatively and significantly correlated (*r* = –0.473, *P* = 0.000, *r* = –0.272, *P* = 0.013) with the MMSE score.

**Table 3:** Correlation between mini-mental state examination score and plasma variables

Variables	All cases (n = 127)		AD (n = 32)		VaD (n = 12)		DOC (n = 83)	
	r value	*P* value	r value	*P* value	r value	*P* value	r value	*P* value
MMSE vs. Age	−0.356	0.000†	0.228	0.210	0.332	0.292	−0.473	0.000†
MMSE vs. Vitamin B12	0.075	0.402	0.231	0.202	−0.226	0.480	0.071	0.523
MMSE vs. Folate	−0.172	0.053	−0.388	0.028*	−0.386	0.215	−0.026	0.812
MMSE vs. TSH	−0.193	0.030*	−0.094	0.609	0.164	0.611	−0.272	0.013*
MMSE vs. TC	−0.010	0.916	−0.055	0.764	0.396	0.203	−0.003	0.976

AD, Alzheimer’s dementia; DOC, Dementia due to other causes; MMSE, Mini-Mental State Examination; TC, Total cholesterol; TSH, Thyroid stimulating hormone; VaD, Vascular dementia. r = Pearson’s correlation coefficients.

* Significant;

† highly significant.

## Discussion

In the present study, we retrospectively tried to find out the possible similarities or differences between AD and VaD and to compare the same in patients with DOC in relation to blood levels of vitamin B_12_, folate, TSH, and TC. There are 2 main findings in our study: One, the levels of vitamin B_12_ and folate were significantly lower in patients with AD and VaD as compared with the controls, but there was no difference in their levels between these 2 groups. Similarly, TSH levels were found to be significantly low in AD and DOC groups as compared with the controls. Two, MMSE score was inversely and significantly related to age, vitamin B_12_, and folic acid. But TSH and TC did not show any correlation with MMSE score in all the 3 groups.

Our first finding, that is, low vitamin B_12_ and folate in AD and VaD patients is compatible with similar studies done in AD and VaD patients.[25,26] However, Koseoglu *et al*. (2007) found that vitamin B_12_ and folate levels were lower significantly in VaD than those in AD.[19] Our findings were further supported by the observation of Clarke *et al*. (1998) who showed that there was a significant association of histologically confirmed AD and VaD with moderately elevated blood levels of homocysteine and with reduced levels of folate and vitamin B_12_.[2] Studies done by Wang *et al*. (2009) also showed that as compared with subjects with normal levels of both the vitamins, subjects with low levels of vitamin B_12_ or folate had double the risk of developing AD.[24] This risk was even stronger in subjects with good cognition at baseline (MMSE score > 26). Biological mechanism proposed to explain the role of low levels of vitamin B_12_ and folate in pathogenesis of AD and VaD is that vitamin B_12_ is necessary for the conversion of homocysteine to methionine and vitamin B_12_ or folate deficiency can increase homocysteine levels due to decreased methylation reaction.[27–29] Hyperhomocysteinemia has also been reported to have a neurotoxic action independent of its vascular effects by overstimulation of *N*- methyl D-aspartate receptors or by an increasing hippocampal neuron vulnerability to excitotoxic insults[30] and amyloid-β peptide toxicity. Furthermore, relative folate deficiency may also exert an adverse effect on the cortical neurons directly.[31] In continuation of our finding, we also found significantly low levels of TSH in AD and DOC groups as compared with the controls. Our findings are in accordance with the studies done by Hoelvorsf *et al*.[32] and Van Osch *et al*.[33] They observed that there is 3-fold increased risk of dementia and AD in persons with reduced TSH levels at baseline. There are several potential explanations for the same. Low TSH levels could be a consequence of AD-related neurodegeneration leading to reduced hypothalamic thyrotrophin-releasing hormone (TRH) secretion or decreased pituitary responsiveness and consequently low TSH levels.[32] Hence TSH levels could precede dementia. The thyroid dysfunction with elevated thyroid hormone levels appears to be associated with increased necrotic neuron death[34] and oxidative stress.[35] Also TRH analogs have been shown to increase acetylcholine synthesis and release in rodents.[36] When this exposure is sustained, acetylcholine depletion may ensure and consequently, the cognitive problems associated with the cholinergic deficit noted in AD brains.

Raised TC observed in all the 3 dementia groups in the present study is supported by the findings of Notkola *et al*. (1998) showing hypercholesterolemia as an independent risk factor for AD.[37] This finding of us further strengthens the increasing lines of evidence suggesting that cholesterol metabolism plays an important role in the pathogenesis of AD.[16] Also, *in vitro* studies support the role of cholesterol in modulating proteolytic processing of amyloid-β precursor protein and/or subsequent amyloid formation and deposition.[38]

Our second finding in this study is as expected, MMSE score was inversely and significantly related to age. We also found that blood levels of vitamin B_12_ and folate also had negative correlation with MMSE score. At present, no effective explanation can be given for the same. The explanation may lie in the limitations or shortfalls of the study. We may put forward 3 reasons: One, vitamin B_12_ and folate levels were measured in our study at the time when patient had developed dementia; second, total vitamin B_12_ levels in the plasma may not signify the deficiency state of vitamin B_12_ at the tissue level; and finally, to establish that such correlation sample size should have been much larger. However, Li *et al*.[16] showed that vitamin B_12_ and folate levels were not related to cognitive function and also found negative correlation between plasma vitamin B_12_ levels and MMSE score in AD patients.

Based on the findings of the present study, it can be concluded that the low levels of these biochemical markers, that is, vitamin B_12_ and folate may be relevant to the clinical course of AD and VaD and should be considered for therapeutic intervention. However, it remains an open question whether or not these interventions in terms of B vitamin supplementation (a combination of folate, vitamin B_12_, and vitamin B_6_) will improve cognitive functions or retard the rate of cognitive decline in older adults with or without dementia. Hence at present, B vitamin supplementation should be reserved for the treatment of documented deficiency states, but not expressly for the prevention or treatment of cognitive disorders, including AD.
